# A set of isogenic auxotrophic strains for constructing multiple gene deletion mutants and parasexual crossings in *Aspergillus niger*

**DOI:** 10.1007/s00203-016-1240-6

**Published:** 2016-06-01

**Authors:** Jing Niu, Mark Arentshorst, Felix Seelinger, Arthur F. J. Ram, Jean Paul Ouedraogo

**Affiliations:** 1 Molecular Microbiology and Biotechnology, Institute of Biology Leiden, Leiden University, Sylviusweg 72, 2333 BE Leiden, The Netherlands; 2 Centre for Structural and Functional Genomics, Concordia University, 7141 Sherbrooke St. W., Montreal, QC H4B 1R6 Canada

**Keywords:** Isogenic strains, Auxotrophy, Multiple markers, Parasexual crossing

## Abstract

To construct a set of isogenic auxotrophic strains in *Aspergillus niger* suited for creating multiple gene deletion mutants and executing parasexual crossings, we have combined mutations in genes involved in colour pigmentation (*fwnA* and *olvA*) with well-selectable auxotrophic markers (*pyrG*, *nicB*, *argB*, and *adeA*). All markers, except for the *pyrG* marker, were introduced by targeted deletion, omitting UV mutagenesis of the strains. *Aspergillus oryzae* orthologous genes of the *argB*, *nicB*, and *adeA* markers were used as heterologous selection markers, and all markers were shown to complement to respective auxotrophic *A. niger* mutants. A quadruple auxotrophic marker was further constructed suitable for multiple gene deletions. Genome sequencing of two auxotrophic colour mutants JN3.2 (*olvA*::*pyrG*, *argB*::*hygB*) and JN6.2 (*olvA*::*pyrG*, *nicB*::*hygB*) revealed four SNPs between them in non-coding regions, indicating a high level of isogenicity between both strains. The availability of near-isogenic complementary auxotrophic colour mutants facilitates the selection of diploids and the isolation of haploid segregants from the diploid using the parasexual cycle.

**Electronic supplementary material:**

The online version of this article (doi:10.1007/s00203-016-1240-6) contains supplementary material, which is available to authorized users.

## Introduction

*Aspergillus niger* has attracted considerable interest as cell factories for the production of organic compounds (citric acid and secondary metabolites) or (recombinant) proteins (Andersen et al. 2013; Meyer et al. 2015; Pel et al. 2007; Ward 2012). *A. niger* is not only an important cell factory, it also has become an important model system for fungal development (Krijgsheld et al. 2013; Wösten et al. 2013). System biology-based approaches in combination with targeted metabolic engineering techniques are important tools to study and optimize production processes (Caspeta and Nielsen 2013; Jacobs et al. 2009). With relative ease, gene knockouts can be made using the *ku70* mutants (Carvalho et al. 2010; Meyer et al. 2007) in combination with split marker approaches (Nielsen et al. 2006; Goswami 2012; Arentshorst et al. 2015a). Together with tools for controlled overexpression of genes using the tetracycline promoter system (Meyer et al. 2011), metabolic engineering can be efficiently performed. A limiting factor for metabolic engineering in *A. niger* is the limited number of isogenic auxotrophic mutants with multiple auxotrophic markers, in which multiple gene deletion mutants can be made quickly without the need to recycle the selection markers. Selection markers such as the *pyrG* marker or the *amdS* marker are counter-selectable, but when multiple deletions need to be made, these markers need to be recycled, which is time-consuming. To overcome this limitation, we have selected the *nicB* gene (encoding nicotinate mononucleotide pyrophosphorylase; Verdoes et al. 1994), the *argB* gene (encoding ornithine carbamoyltransferase; Lenouvel et al. 2002), and the *adeA* gene (encoding phosphoribosylaminoimidazole-succinocarboxamidesynthase) (Jin et al. 2004; Ugolini and Bruschi 1996) of *A. niger* to construct near-isogenic auxotrophic marker strains containing four auxotrophic markers (*pyrG*, *nicB*, *adeA*, and *argB*). In combination with dominant selection markers such as hygromycin resistance (Punt and van den Hondel 1992), phleomycin resistance (Punt and van den Hondel 1992), and AmdS selection (Kelly and Hynes 1985), seven different markers are available for strain construction.

The lack of a sexual cycle in *A. niger* limits easy crossing of two strains to combine interesting properties or to construct double mutants. Despite the lack of a sexual cycle, the parasexual cycle can be used to combine genetic traits in *A. niger* (Pontecorvo et al. 1953; Swart et al. 2001). The parasexual cycle includes the selection of a heterokaryon and subsequently the selection of a diploid strain. The frequency by which diploids are formed from a heterokaryotic mycelium in *A. niger* is very low, and selection of diploids can be accomplished by crossing strains that have complementary auxotrophic and complementary spore colour markers. Only when a diploid is formed, the resulting colony will produce solely black conidiospores which can be easily detected by eye. The genes encoding proteins involved in spore melanin production in *A. niger* have been identified (Jørgensen et al. 2011). Several studies, mainly conducted by Bos et al., have reported on the isolation of *A. niger* colour and auxotrophic mutants [see for review (Swart et al. 2001)]. However, most of these mutants were isolated by UV treatment. Although carried out with caution and relative high survival rates, unwanted random mutations are inevitable, leading to possible growth defects. By targeted deletion of spore colour genes and auxotrophies, we constructed a set of near-isogenic strains suitable for parasexual crossings (Niu et al. 2016). We performed genome sequencing of two auxotrophic colour mutants and confirmed the near-isogenicity between these auxotrophic mutants.

## Materials and methods

### Strains and growth conditions

The *A. niger* strains used in this study are listed in Table 1. Auxotrophic strains are deposited at the Fungal Genetic Stock Centre. *A. niger* strains were grown on minimal medium (MM) (Bennet and Lasure 1991) or on complete medium (CM) consisting of minimal medium with the addition of 5 g/L yeast extract and 1 g/L casamino acids. When required, 10 mM uridine, 200 μg/mL L-arginine, 2.5 μg/mL nicotinamide, 100 μg/mL hygromycin, or 40 μg/mL phleomycin was added. Adenine was directly added from the solid stock to the medium to a final concentration of 200 mg/L after autoclaving and dissolved by mixing. Fluoroacetamide (FAA) and 5-fluoroorotic acid (5-FOA) counter-selection was performed as described (Carvalho et al. 2010; Arentshorst et al. 2012) to remove the *amdS* marker and the *pyrG* marker, respectively.

**Table 1:** Strains used in this study

Name	Genotype/description	Reference/source
N402	*cspA1*, derivative of N400	Bos et al. (1988)
*A. oryzae*	ATCC16868	–
MA169.4	*kusA*::*amdS*, *pyrG* ^*−*^	Carvalho et al. (2010)
MA100.1	*cspA1*, *fwnA*::*hygB*, *kusA*::*amdS*, *pyrG* ^*−*^	Jørgensen et al. (2011)
AW8.4	*cspA1*, *olvA*::*AopyrG* in MA169.4	Jørgensen et al. (2011)
JN3.2	*argB*::*hygB*, *olvA*::*AopyrG* (derived from AW8.4)	This study
JN6.2	*nicB*::*hygB*, *olvA*::*AopyrG* (derived from AW8.4)	This study
JN1.17.1	*argB*::*hygB* in MA169.4	This study
OJP3.1	*nicB*::*phleo* in MA169.4	This study
OJP1.1	*adeA*::*pyrG* in MA169.4	This study
MA322.2	*ku70*::*amdS*, *nicB*::*AopyrG* in MA169.4	This study
MA323.1	*ku70*::*amdS*, Δ*nicB* ^*−*^, *pyrG* ^*−*^	This study
MA328.2	*ku70*::*amdS*, Δ*nicB* ^*−*^, *adeA*::*AopyrG*	This study
MA329.1	*ku70*::*amdS*, Δ*nicB* ^*−*^, Δ*adeA* ^*−*^, *pyrG* ^*−*^	This study
MA334.2	*ku70*::*amdS*, Δ*nicB* ^*−*^, Δ*adeA* ^*−*^, *argB*::*AopyrG*	This study
MA335.3	*ku70*::*amdS*, Δ*nicB* ^*−*^, Δ*adeA* ^*−*^, Δ*argB* ^*−*^, *pyrG* ^*−*^	This study

### Molecular biological techniques

Transformation of *A. niger* and chromosomal DNA isolation of *A. niger* and *Aspergillus oryzae* were performed according to (Meyer et al. 2010). Southern blot analysis was performed according to (Sambrook and Russell 2001). α-^32^P-dCTP-labelled probes were synthesized using the Rediprime II kit (Amersham, GE Healthcare), according to the instructions of the manufacturer. Restriction and ligation enzymes were obtained from Thermo Scientific and used according to the instructions of the manufacturer. PCR was performed with Phire Hot Start II DNA polymerase or Phusion DNA polymerase (Thermo Scientific). Sequencing was performed by Macrogen.

### Construction of plasmids and deletion cassettes

The deletion cassettes for the *argB*, *nicB*, and *adeA* genes of *A. niger* were constructed with the *hygB*, *phleo*, and *pyrG* selection markers, respectively. The plasmid used to disrupt the *argB* gene (An14g03400) with the hygromycin selection marker was constructed as follows: ~0.8-kb DNA fragments flanking the *argB* ORF were amplified by PCR using N402 genomic DNA as template, with primers listed in Supplementary Table 1. The PCR products were cloned into pJet1.2 (Thermo Scientific). The 5′flank of *argB* was excised from pJet1.2 using *Kpn*I/*Hind*III and inserted into the same site of pBlueScript II Sk(+) to obtain plasmid pJN3.3. Subsequently, pJN3.3 was digested with *Hind*III/*Not*I and used in a three-way ligation with the 3′flank of *argB* excised from pJet1.2 using *Xho*I/*Not*I and the 3-kb *Hind*III/*Xho*I fragment containing the *hygB* gene, obtained from plasmid pΔ2380 (Damveld et al. 2008), resulting in the *argB* disruption plasmid pJN4.5. The *argB* gene deletion cassette was amplified by PCR using pJN4.5 DNA as template with primers argBKO1 and argBKO4 and the purified linear PCR fragment was used for subsequent transformation to *A. niger* strain MA169.4 (*ku70*^*−*^, *pyrG*^*−*^) to give JN1.17.1 (*ku70*^*−*^*, pyrG*^*−*^, Δ*argB*::*hygB*) or to *A. niger* strain AW8.4 (*ku70*^*−*^, Δ*olvA*::*AOpyrG*), resulting in JN3.2 (*ku70*^*−*^, Δ*olvA*::*AOpyrG*, Δ*argB*::*hygB*).

The same approach was used to construct the disruption cassettes of the *nicB* gene (An11g10910) of *A. niger* with either the phleomycin or hygromycin marker. The DNA fragments flanking the *nicB* ORF were amplified from N402 genomic DNA, with primers listed in Supplementary Table 1. After cloning in pJet1.2, the 5′flank of *nicB* was isolated as a *Kpn*I/*Xho*I fragment and inserted into *Kpn*I/*Xho*I-opened pBlueScript II SK(+) to obtain plasmid pJN8.1. Subsequently, the 1.9-kb *Xho*I–*Hind*III fragment containing *phleo* expression cassette, obtained from plasmid pMA299, or the 3.1-kb *Xho*I–*Hind*III fragment containing *hygB* expression cassette, obtained from plasmid pΔ2380 (Damveld et al. 2008), together with the *Hind*III/*Not*I isolated 3′flank of *nicB*, were ligated into *Xho*I/*Not*I-opened pJN8.1, resulting in the *nicB*::*phleo* disruption plasmid pJN10.1 or *nicB*::*hygB* disruption plasmid pJN9.1. The *nicB* gene deletion cassettes were amplified by PCR using pJN10.1 or pJN9.1 as template with primer NicBKO1 and NicBKO4 and used for transformation to *A. niger* strain MA169.4 (*ku70*^*−*^, *pyrG*^*−*^) to give OJP3.1 (*ku70*^*−*^, *pyrG*^*−*^, Δ*nicB*::*phleo*) or to *A. niger* strain AW8.4 (*ku70*^*−*^, Δ*olvA*::*AOpyrG*), resulting in JN6.2 (*ku70*^*−*^, Δ*olvA*::*AOpyrG*, Δ*nicB*::*hygB*).

To construct the disruption cassette of *adeA* gene (An11g10150), the flanking regions of the gene were amplified by PCR from N402 genomic DNA with primers Fw_adeA_5′ and Rev_adeA_5′ to obtain the 0.9-kb 5′flanking region and Fw_adeA_3′and Rev_adeA_3′ to obtain the 0.7-kb 3′flanking region (Supplementary Table 1). The 1.8-kb *A. nidulanspyrG* selection marker was amplified by PCR from the plasmid *pCRpyrGAN* (Ouedraogo et al. 2015) with the primers Fw_pyrG_adeA and Rev_pyrG_adeA which contain complementary sequence of Rev_adeA-5′and Fw_adeA-3′, respectively (Supplementary Table 1). The *adeA*::*Anid_pyrG* deletion cassette was obtained by a fusion PCR of the three purified PCR products, followed by cloning of the 3.4-kb fusion PCR product into pJet1.2, resulting in plasmid pOJP1 and used for transformation to *A. niger* strain MA169.4 (*ku70*^*−*^, *pyrG*^*−*^) to give OJP1.1 (*ku70*^*−*^, Δ*adeA*::*pyrG*). Proper deletion of the *nicB*, *adeA*, and *argB* genes was confirmed by Southern blot analysis (Supplementary Figures. 1–3).

For complementation studies, *argB*, *nicB*, and *adeA* genes, including their promoter and terminator regions, were amplified from wild-type *A. oryzae* and *A. niger* genomic DNA with appropriate primer pairs described in the Supplementary Table 1. The respective complementing gene fragments were cloned into pJet1.2 (Thermo Scientific) and sequenced (Table 2). The plasmids pOJP5 (pJet1.2_*Anig.argB*), pOJP4 (pJet1.2_*Anig.nicB*), pOJP3 (pJet1.2_*Anig.adeA*), pJN29 (pJet1.2_*Aory.argB*), pJN30 (pJet1.2_*Aory.nicB*), and pJN31 (pJet1.2_*Aory.adeA*) were used to complement the respective auxotrophic mutants.

**Table 2:** Plasmids used in this study

Name	Description	Reference/source
pJN3.3	5′flank of *argB* in pBluescript II SK(+)	This study
pΔ2380	Δ*ugmB*::*hygB* deletion cassette	Damveld et al. (2008)
pJN4.5	pBluescript_*argB*::*hygB*	This study
pJN8.1	5′flank of *nicB* in pBluescript II SK(+)	This study
pMA299	pBluescript_*phleo*	This study
pJN10.1	pBluescript_*nicB*::*phleo*	This study
pCRpyrGAN	Containing the full gene of *A. nidulans* *pyrG*	Ouedraogo et al. (2015)
pOJP1	pJet1.2_*adeA*::*pyrG*	This study
pOJP5	pJet1.2_*Anig.argB*	This study
pOJP4	pJet1.2_*Anig.nicB*	This study
pOJP3	pJet1.2_*Anig.adeA*	This study
pJN29	pJet1.2_*Aory.argB*	This study
pJN30	pJet1.2_*Aory.nicB*	This study
pJN31	pJet1.2_*Aory.adeA*	This study
pAO4-13	Containing full *pyrG* gene of *A. oryzae*	de Ruiter-Jacobs et al. (1989)

### Recyclable split marker strategy for creation of a strain with multiple auxotrophies

**Fig. 1:**
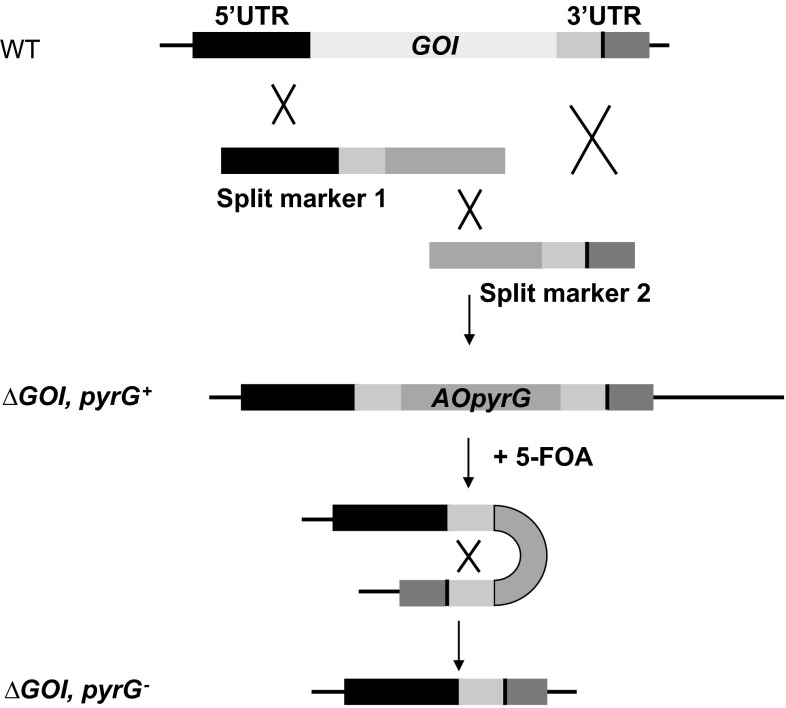
Schematic representation of the recyclable split marker approach for multiple gene deletion mutants. Deletion of the gene of interest (GOI) by split marker approach with recycling of the *Aspergillus oryzae pyrG* marker. The split marker fragments 1 and 2 are used during transformation to knock out the GOI by homologous recombination which generates a uridine prototroph (*pyrG* ^+^) strain. The *pyrG* marker is subsequently looped out by 5-FOA selection, and the resulting *pyrG* ^*−*^ strain is suitable for a second gene deletion with the *pyrG* marker. The split marker approach is described previously (Arentshorst et al. 2015a)

To construct an *A. niger* strain with multiple auxotrophies, it was necessary to use a recyclable split marker approach. Therefore, auxotrophic marker-specific direct repeats (DR) surrounding the *AOpyrG* selection marker were introduced by PCR. By selecting on 5-FOA, the *AOpyrG* marker was removed. The recyclable split marker approach is outlined in Fig. 1; see Supplementary Table 1 for primer sequences. Strain MA169.4 (*ku70*^*−*^, *pyrG*^*−*^) was used as starting strain to first delete the *nicB* gene and, subsequently, *adeA* and the *argB* marker. All strains containing single, double, triple, and the quadruple auxotrophic strain are listed in Table 1. Correct integration of split marker fragments and successful loop out of the *AOpyrG* was confirmed by Southern blot analysis for all strains and shown for MA335.3 in Supplementary Figures. 1–3).

### *A. niger* parasexual cycle

Heterokaryon formation and selection for diploids was performed as described (Pontecorvo et al. 1953). Segregation of diploids by benomyl was performed essentially as described (Bos et al. 1988) with slight modifications (Niu et al. 2016).

### Sequencing and analysis

Genome sequencing of JN3.2 (*olvA*::*pyrG*, *argB*::*hygB*) and JN6.2 (*olvA*::*pyrG*, *nicB*::*hygB*) was performed using NGS platform (Illumina GA) as described (Park et al. 2014). Sequencing was performed at ServiceXS, Leiden, The Netherlands. SNPs between JN3.2 and JN6.2 were identified using *A. niger* strain ATCC1015 (http://genome.jgi-psf.org/pages/search-for-genes.jsf?organism=Aspni5) as reference genome. For each SNP, it was verified whether the SNP was in a predicted protein-encoding region using the *A. niger* 3.0 genome at JGI using the SNP coordinates (Park et al. 2014).

## Results and discussion

### Construction and characterization of *argB*, *nicB*, and *adeA* auxotrophic mutants

**Fig. 2:**
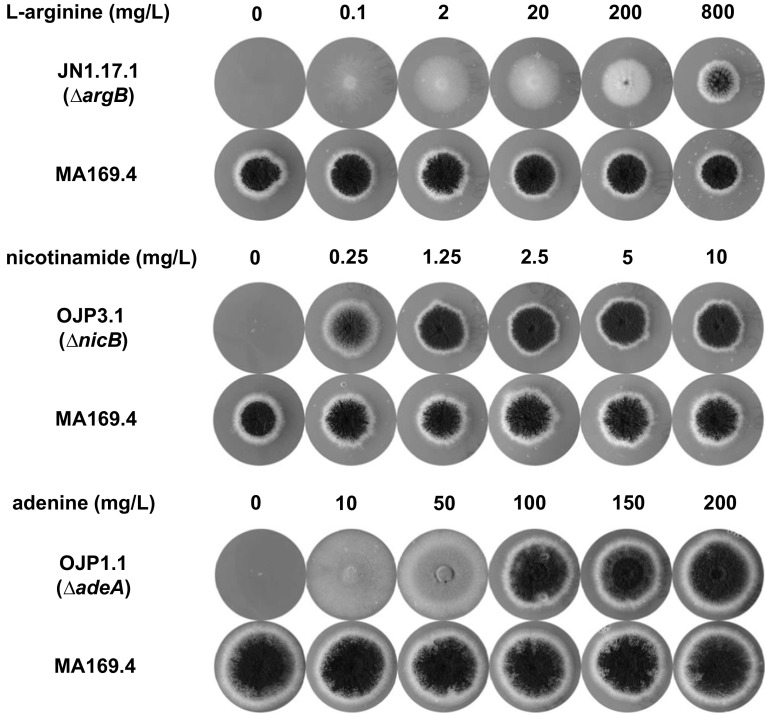
Supplementation test of the auxotrophic *A. niger* mutants. 10 µL of a spore stock (1 × 10^7^ conidia/mL) of each auxotrophic strain and the parental strain (MA169.4) was inoculated on an MM plate without and with serial concentrations of the respective supplement and incubated at 30 °C for 3 days for arginine and nicotinamide supplementation test and for 4 days for adenine supplementation test

Deletion constructs *nicB*::*hygB*, *argB*::*phleo*, and *adeA*::*pyrG* were transformed to strain MA169.4 (*ku70*^*−*^*, pyrG*^*−*^), and hygromycin, phleomycin resistant, or uridine prototrophic transformants were obtained and purified. Proper deletion of the respective markers was verified by diagnostic PCRs (data not shown) and by testing the growth on MM plates containing the relevant supplements. As shown in Fig. 2, the *nicB*, *argB*, and *adeA* mutants required the addition of the nicotinamide, l-arginine, or adenine to allow growth.

To determine the minimal concentrations of nicotinamide, arginine, or adenine for full supplementation, spores of the auxotrophic mutants were spotted on plates containing a concentration series of the respective supplements and the growth was monitored over time. The results in Fig. 2 show the necessity to use at least 800 mg/L of arginine and 1.25 mg/L of nicotinamide to fully supplement the ∆*argB* and ∆*nicB* strains, respectively. For the ∆*adeA* mutant, the supplementation test shows that a concentration of adenine between 10 and 50 mg/L leads to the accumulation of red pigment. At this range of adenine concentrations, the strain is not forming conidia. Further analysis showed that this red pigment was accumulated into the vacuole when cells were grown in liquid medium (data not shown). To fully supplement the ∆*adeA* mutant, at least 150 mg/L of adenine in the growth medium was required.

### Construction and characterization of a quadruple auxotrophic strain (∆*nicB*, ∆*argB*, ∆*adeA*, *pyrG*^*−*^)

**Fig. 3:**
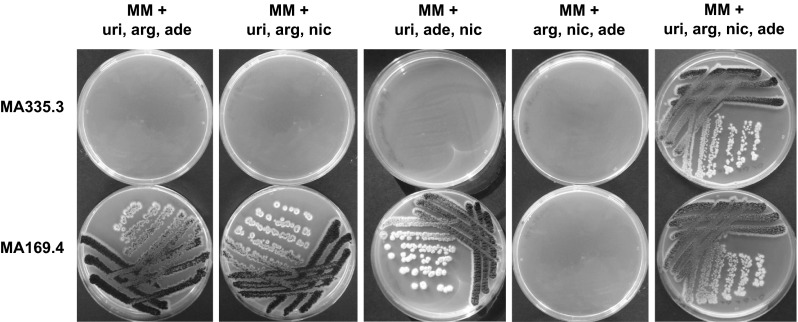
Growth analysis of the quadruple auxotrophic *A. niger* strain. MA335.3 (∆*nicB*, ∆*argB*, ∆*adeA*, *pyrG* ^*−*^) was plated on solid MM with and without the different supplements at 30 °C, and growth was analysed after 3 days. The parental strain MA169.4 was taking along the analysis for comparison

We have constructed a quadruple auxotrophic strain based on the recyclable split marker approach described in Fig. 1 and in materials and methods. This approach allows iterative construction of gene knockouts in *A. niger* by subsequent recycling of the *pyrG* marker using counter-selection on 5-FOA, due to the presence of the direct repeated sequences flanking the selection marker. The proper deletion and absence of ectopic copies of the deletion cassettes in the quadruple auxotrophic strain MA335.3 was confirmed by Southern blot analysis (Supplemental Figures. 1–3) and characterized by the inability to growth in the absence of arginine, nicotinamide, adenine, or uridine (Fig. 3). This quadruple auxotrophic strain offers the possibility to delete multiple genes without the need to recycle the selection marker.

### The *nicB*, *argB*, and *adeA* genes from *A. oryzae* are suitable markers for *A. niger* transformation

**Fig. 4:**
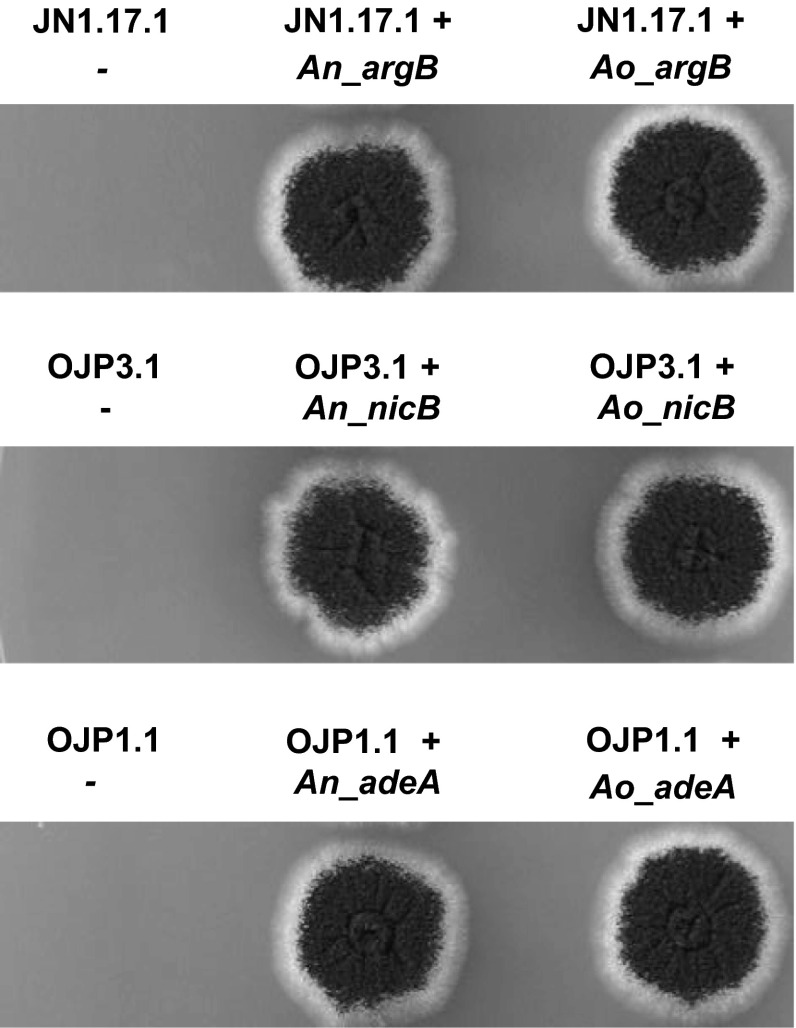
Growth analysis of the complemented transformants. Spores of JN1.17.1 (Δ*argB*, *pyrG* ^*−*^) OJP3.1 (Δ*nicB*, *pyrG* ^*−*^) and OJP1.1 (Δ*adeA*) and complemented strains were spotted on selective medium to test complementation of the *argB*, *nicB*, and *adeA*, respectively, from *A. niger* (An) or *A. oryzae* (Ao). Pictures were taken after 3 days of growth at 30 °C

To prove that auxotrophic mutants can be complemented by heterologous and homologous markers, DNA fragments containing the *argB*, the *nicB*, and the *adeA* genes from *A. oryzae* and *A. niger*, including their promoters and 3′ untranslated sequences, were used for the complementation of the respective *A. niger* auxotrophic mutants. Protoplasts of JN1.17.1 (Δ*argB*::*hygB*), OJP3.1 (Δ*nicB*::*phleo*), and OJP1.1 (Δ*adeA*::*pyrG*) were transformed with plasmids containing the corresponding marker genes from *A. oryzae* or *A. niger*. Transformants were obtained for the *A. oryzae* heterologous markers, which demonstrated that *nicB, argB*, and *adeA* of *A. oryzae* complemented the auxotrophy and therefore are suitable markers for *A. niger* transformations. As expected, also all *A. niger* genes (*argB*, *nicB*, and *adeA*) were able to complement the respective auxotrophic *A. niger* mutants. The obtained transformants were further analysed to determine whether the *A. oryzae* marker also complemented the auxotrophies. As shown in Fig. 4, all heterologous genes complement similarly to the homologous *A. niger* genes. A heterologous marker for gene disruption experiments is preferred as it reduces the homologous integration of the marker gene in the disruption cassette at the homologous site. We have compared the DNA sequence of the different genes markers of *A. niger* to those of *A. oryzae* by BLASTN (http://blast.ncbi.nlm.nih.gov/) using standard settings. The identity of the coding regions between the different gene markers was 73.3, 72.0, and 77.8 % for *argB*, *nicB*, and *adeA* genes, respectively. These values are comparable to the value obtained when comparing the *pyrG* genes markers of both *Aspergillus* species. The *pyrG* gene of *A. oryzae* is identical to the *pyrG* gene of *A. niger* at 78.6 % and has been so far successfully used to transform *A. niger* and vice versa (Carvalho et al. 2010; Mattern et al. 1987). It should be noted that complementation analysis in the ∆*ku70* background is not efficient because of the low frequencies of ectopic integration the complementing fragment. To circumvent this limitation, we constructed a curable *ku70* deletion strategy (Carvalho et al. 2010). The presence of *ku70* repeats around the AmdS selection marker used to disrupt the *ku70* gene allows efficient loop out of the AmdS marker via fluoroacetamide counter-selection as described (Arentshorst et al. 2012). An alternative method for easy complementation, which omits the need for curing the *ku70* locus, is the use of a second auxotrophic marker which can be used to target the complementing gene to this locus. For the *pyrG* marker, an efficient gene targeting method has recently become available (Arentshorst et al. 2015b) which allows targeted integration when the complementing fragment is cloned in the *pyrG* targeting vector. For example, one could start with a *nicB*^*−*^, *pyrG*^*−*^ strain and use the *nicB* selection marker for initial deletion of the gene of interest, followed by a complementation experiment in which the complementing fragment is cloned in the *pyrG* targeting vector which is that transformed to the deletion strain.

### Isogenic auxotrophic colour mutants for parasexual crossing in *A. niger*

Combining mutations by crossing strains is a powerful genetic tool for strain construction. In *Aspergillus nidulans,* this method is well established and used in many studies to construct double mutants (Todd et al. 2007). The lack of a sexual cycle in *A. niger* has limited the use of crossings to combine mutations. However, the use of the parasexual cycle in *A. niger* (Pontecorvo et al. 1953) has been used extensively for linkage studies in *A. niger* and can be used to combine mutations (Bos et al. 1988). Straightforward crossing in *A. niger* requires complementing auxotrophies to select for a heterokaryotic mycelium and preferably colour makers to select for a diploid strain. The frequency by which *A. niger* forms diploids is generally very low (1 in 10^6^–10^7^ spores), and diploids are not easily detected if wild-type strains are used that produce black conidia. By using complementing colour markers, a diploid can be selected as only this diploid will produce black spores, whereas a heterokaryotic mycelium will produce a mix of heterogeneously coloured spores (Pontecorvo et al. 1953). By combining colour mutants (*fwnA* and *olvA*) with complementary auxotrophic markers such as *pyrG*, *nicB*, or *argB*, heterokaryons and diploids can be easily selected. We constructed several auxotrophic colour mutant strains including MA100.1 (*fwnA*::*hygB*, *pyrG*^*−*^), JN3.2 (*olvA*::*pyrG*, *argB*::*hygB*), and JN6.2 (*olvA*::*pyrG*, *nicB*::*hygB*) (Table 1). In a recently conducted study, JN3.2 has been used for parasexual crossings to obtain haploid segregants (Niu et al. 2016). With these segregants, a bulk segregant analysis was performed to identify SNPs that are closely linked or responsible for the mutant phenotypes (Niu et al. 2016).

To test the isogenicity between two auxotrophic colour mutants JN3.2 (*olvA*::*pyrG*, *argB*::*hygB*) and JN6.2 (*olvA*::*pyrG*, *nicB*::*hygB*), the genomes of these strains were sequenced and compared to the genome of the reference ATCC strain. In total, 155 SNPs were found for JN3.2 and JN6.2, respectively, when compared to the ATCC reference strain (Supplementary Table 2). Two SNPs were found to be specific for JN3.2, and two SNPs were specific for JN6.2. None of them were found in predicted open reading frames (Table 3), demonstrating that JN3.2 and JN6.2 are likely to have no mutation affected its phenotype and that they are near-isogenic.

**Table 3:** SNP comparison JN6.2 and JN3.2

	Position	Allel ATCC	JN6.2	JN3.2	Details mutation
chr_1_2	726,573	T	T	C	Intergenic
chr_3_4	45,864	T	T	A	Intergenic
chr_8_2	2,725,044	G	A	G	Intergenic
chr_8_2	2,725,045	T	A	T	Intergenic

In conclusion, new auxotrophic strains carrying targeted deletions in the *argB*, *nicB*, and *adeA* genes of *A. niger* were constructed. The orthologous genes *argB*, *nicB*, and *adeA* of *A. oryzae* complemented the arginine, nicotinamide, and adenine auxotrophic mutants similar to the endogenous genes and are therefore suitable selection markers for *A. niger* transformations. The quadruple auxotrophic strain MA335.3 (*argB*^*−*^, *nicB*^*−*^, *adeA*^*−*^, and *pyrG*^*−*^) allows rapid deletion of multiple genes the need to recycle selection markers. The targeted deletion of auxotrophic markers instead of selection of auxotrophic strains after UV mutagenesis significantly reduces the occurrence of mutations as genome sequencing of two auxotrophic mutants (JN3.2 and JN6.2) revealed only four SNP between them.

## Electronic supplementary material

Below is the link to the electronic supplementary material.

Supplementary material 1 (DOCX 24 kb)

Supplementary material 2 (XLSX 28 kb)

Supplemental Fig. 1. Verification of the *nicB* deletion in OJP3.1 (*nicB*::*phleo* in MA169.4) and quadruple auxotrophic strain MA335.3. A) Schematic representation of the *nicB* locus of the wild type and the *nicB*::*phleo* deletion strain and after loop out of the *pyrG*. Predicted sizes of the DNA fragment hybridizing with the indicated probe are shown. B) Southern blot analysis of genomic DNA of MA169.4 (lane 1), OJP3.1 (lane 2), MA335.3. (lane 3), and MA335.4 (lane 4). Left panel: agarose gel stained with ethidium bromide. MW = molecular weight marker size (in kb) is indicated. Right panel: Southern blot after hybridization with *nicB* probe. (PPTX 228 kb)

Supplemental Fig. 2. Verification of the *adeA* deletion in OJP1.1 (*adeA*::*pyrG* in MA169.4) and quadruple auxotrophic strain MA335.3. A) Schematic representation of the *adeA* locus of the wild type and the *adeA*::*pyrG* deletion strain and after loop out of the *pyrG*. Predicted sizes of the DNA fragment hybridizing with the indicated probe are shown. B) Southern blot analysis of genomic DNA of MA169.4 (lane 1), MA335.3 (lane 2), MA335.4 (lane 3), and OJP1.1 (lane 4). Left panel: agarose gel stained with ethidium bromide. MW = molecular weight marker size (in kb) is indicated. Right panel: Southern blot after hybridization with *adeA* probe. (PPTX 228 kb)

Supplemental Fig. 3. Verification of the *argB* deletion in JN1.17.1 (*arg*::*hygB* in MA169.4) and quadruple auxotrophic strain MA335.3. A) Schematic representation of the *argB* locus of the wild type and the *argB*::*hygB* deletion strain and after loop out of the *pyrG* gene. Predicted sizes of the DNA fragment hybridizing with the indicated probe are shown. B) Southern blot analysis of genomic DNA of MA169.4 (lane 1), JN1.17.1 (lane 2), MA335.3 (lane 3), and MA335.4 (lane 4). Left panel: agarose gel stained with ethidium bromide. MW = molecular weight marker size (in kb) is indicated. Right panel: Southern blot after hybridization with *argB* probe. (PPTX 243 kb)
