# Supplementary material for: A set of isogenic auxotrophic strains for constructing multiple gene deletion mutants and parasexual crossings in Aspergillus niger
Source: Arch Microbiol. 2016 Jun 1;198(9):861–8. doi: 10.1007/s00203-016-1240-6 (PMC5040738; doi:10.1007/s00203-016-1240-6)
Supplement: Supplementary file 1 — Supplementary material 1 (DOCX 24 kb) [file 203_2016_1240_MOESM1_ESM.docx]

article title: A set of isogenic auxotrophic strains for constructing multiple gene deletion mutants and parasexual crossings in Aspergillus niger

journal name: Archives of Microbiology

author names: Jing Niu, Mark Arentshorst, Felix Seelinger, Arthur F.J. Ram*, Jean Paul Ouedraogo

affiliation: Molecular Microbiology and Biotechnology, Leiden University, Sylviusweg 72, 2333 BE Leiden, the Netherlands

corresponding author : a.f.j.ram@biology.leidenuniv.nl

**Supplemental Table 1**. Primers used in this study.

| **Primer name** | **f/r** | **Sequence 5’ to 3’** | **Template** |
| --- | --- | --- | --- |
| argBKO1 | f | GGTACCAATGATGTCAGCTACGAACGG | N402 |
| argBKO2 | r | AAGCTTGGGATTGGGAGGTAATGGTAGA | N402 |
| argBKO3 | f | CTCGAGGAGTAATTGGATGGGTGCATTT | N402 |
| argBKO4 | r | GCGGCCGCGAGACCGAGTTTCAACGGGT | N402 |
| argBCheck1 | f | TTGGCTGGTGTGCTCAAGC | argB- transformants |
| argBCheck2 | r | CTGAATTCCCAGATGCTGTTG | argB- transformants |
| NicBKO1 | f | GGTACCCCCAAGACCAAACAAGGCAGTG | N402 |
| NicBKO2 | r | CTCGAGTACGAAGCCGCCATAGTCG | N402 |
| NicBKO3 | f | AAGCTTGGTTGAGTGTCGGAGTTTGGA | N402 |
| NicBKO4 | r | GCGGCCGCTTCTTTCTTGGACATTTGCCG | N402 |
| NicBCheck1 | f | AAATTGTGCTGCCTGTAGTTCAC | nicB- transformants |
| NicBCheck2 | r | ATATTGGGCGTTCCAGGGC | nicB transformants |
| Fw_adeA_5’ | f | TCCGCAGACTGTCTTGGATG | N402 |
| Rev_adeA_5’ | r | GGGTGGAATGGAGACAATGG | N402 |
| Fw_adeA_3’ | f | CTCTGACTCTGTCCCTTACC | N402 |
| Rev_adeA_3’ | r | TCCTAAGAACTGGCGCGTTG | N402 |
| Fw_pyrG_adeA | f | CCATTGTCTCCATTCCACCCTCGCCCTTGCTCTAGATAAC | pCRpyrGAN |
| Rev_pyrG_adeA | r | GGTAAGGGACAGAGTCAGAGTAATTCGCCCTTGACTAGTGC | pCRpyrGAN |
| Fw_adeAHR | f | CACGGTATATTCGTACGACG | adeA- transformants |
| Rev_adeAHR | r | CCATGGAAGTATCTCGAC | adeA- transformants |
| Fw_argB_nig | f | GGTACCAATGATGTCAGCTACGAAGG | pOJP5 |
| Rev_argB_nig | r | GCGGCCGCGAGACCGAGTTTCAACGGGT | pOJP5 |
| Fw_nicB_nig | f | ACATCCATCGTGTCGCAAAC | pOJP4 |
| Rev_nicB_nig | r | AGAACCAGGCTCTTTGATCC | pOJP4 |
| Fw_adeA_nig | f | TCCGCAGACTGTCTTGGATG | N402 and pOJP3 |
| Rev_adeA_nig | r | TCCTAAGAACTGGCGCGTTG | N402 and pOJP3 |
| Fw_argB_ory | f | GATTGAATACGGTGGCATCC | pJN29 |
| Rev_argB_ory | r | CAGGTGATTCCACGTCATAG | pJN29 |
| Fw_nicB_ory | f | GGCTACTCAACAGCACTAAG | pJN30 |
| Rev_nicB_ory | r | CGGATTATTCTGGCGACAAC | pJN30 |
| Fw_adeA_ory | f | CTACATTTGGCCCTTGTCTG | pJN31 |
| Rev_adeA_ory | r | GGTACGGTTACCTTTCCATC | pJN31 |
| nicBP1f | f | GCAGTGCACA TCCATCGTG | N402 |
| nicBP2r | r | CACATCATAAAGATCCACCCATGTTGGATGTAGTGATGTTGTTG | N402 |
| nicBP3f | f | TGGGTGGATCTTTATGATGTG | N402 |
| nicBP4f | f | ACACGGCACAATTATCCATCGTGGGTGGATCTTTATGATGTG | N402 |
| nicBP5r | r | CAATTCCAGCAGCGGCTTGAGGTTTGAGCCTAGCCTTGG | N402 |
| nicBP6r | r | AACCAGGCTCTTTGATCCGTC | N402 |
| adeAP1f | f | CGTCGCAAGTCTGAAAGCATT | N402 |
| adeAP2r | r | CAGTCCACCAGGACCTGATAATGTTTGGGGTGGAATGGAGAC | N402 |
| adeAP3f | f | TTATCAGGTCCTGGTGGACTG | N402 |
| adeAP4f | f | ACACGGCACAATTATCCATCGTTATCAGGTCCTGGTGGACTG | N402 |
| adeAP5r | r | CAATTCCAGCAGCGGCTTGACGTCGATCATTCGGCAG | N402 |
| adeAP6r | r | TTCCTAAGAACTGGCGCGTT | N402 |
| argBP1f | f | CTGGCAATGGCTGCAATTC | N402 |
| argBP2r | r | CTACTCAAAATGCACCCATCCAATGTGAGTTTGGTAGGAGGGATTG | N402 |
| argBP3f | f | TTGGATGGGTGCATTTTGAGTAG | N402 |
| argBP4f | f | ACACGGCACAATTATCCATCGTTGGATGGGTGCATTTTGAGTAG | N402 |
| argBP5r | r | CAATTCCAGCAGCGGCTTGGGCTGTGATGGAACTGATTG | N402 |
| argBP6r | r | CGGGTGTTGCAGTCTAAGGG | N402 |
| AOpyrGP12f | f | AAGCCGCTGCTGGAATTG | pAO4-13 |
| AOpyrGP13r | r | CGATGGATAATTGTGCCGTGT | pAO4-13 |
| AOpyrGP14f | f | ATTGACCTACAGCGCACGC | pAO4-13 |
| AOpyrGP15r | r | CCGGTAGCCAAAGATCCCTT | pAO4-13 |
