# Supplementary material for: A set of isogenic auxotrophic strains for constructing multiple gene deletion mutants and parasexual crossings in Aspergillus niger
Source: Arch Microbiol. 2016 Jun 1;198(9):861–8. doi: 10.1007/s00203-016-1240-6 (PMC5040738; doi:10.1007/s00203-016-1240-6)
Supplement: Supplementary file 5 — Supplemental Fig. 3. Verification of the argB deletion in JN1.17.1 (arg::hygB in MA169.4) and quadruple auxotrophic strain MA335.3. A) Schematic representation of the argB locus of the wild type and the argB::hygB deletion strain and after loop out of the pyrG gene. Predicted sizes of the DNA fragment hybridizing with the indicated probe are shown. B) Southern blot analysis of genomic DNA of MA169.4 (lane 1), JN1.17.1 (lane 2), MA335.3 (lane 3), and MA335.4 (lane 4). Left panel: agarose gel stained with ethidium bromide. MW = molecular weight marker size (in kb) is indicated. Right panel: Southern blot after hybridization with argB probe. (PPTX 243 kb) [file 203_2016_1240_MOESM5_ESM.pptx]

## Slide 1
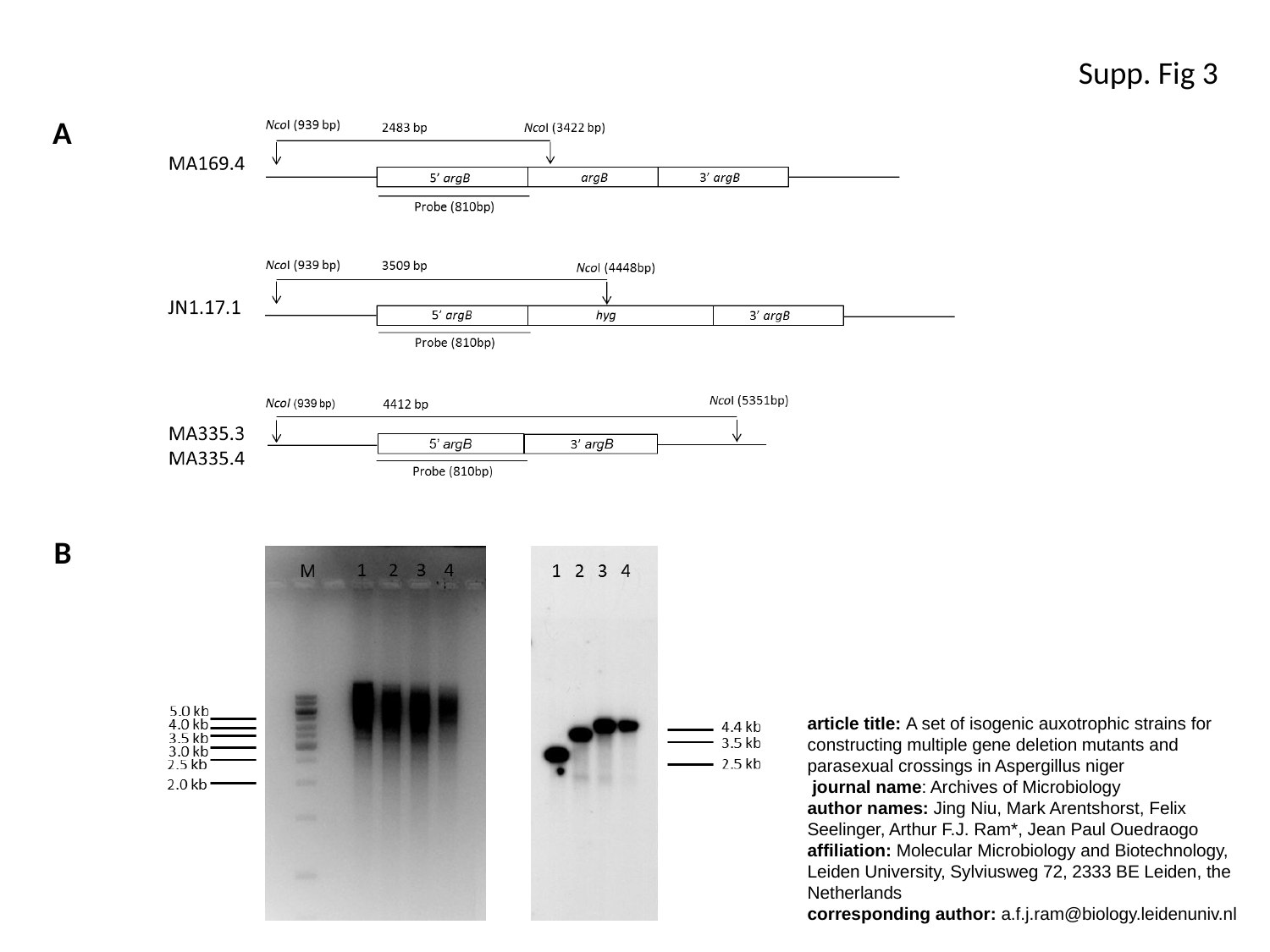

Supp. Fig 3
A
B
article title: A set of isogenic auxotrophic strains for constructing multiple gene deletion mutants and parasexual crossings in Aspergillus niger
 journal name: Archives of Microbiology
author names: Jing Niu, Mark Arentshorst, Felix Seelinger, Arthur F.J. Ram*, Jean Paul Ouedraogo
affiliation: Molecular Microbiology and Biotechnology, Leiden University, Sylviusweg 72, 2333 BE Leiden, the Netherlands
corresponding author: a.f.j.ram@biology.leidenuniv.nl
